# Navigation-guided elective primary total hip arthroplasty is associated with reduced 90-day readmission and early revision at increased index cost: a national analysis

**DOI:** 10.3389/fsurg.2026.1818308

**Published:** 2026-08-17

**Authors:** David Maman, Yaniv Steinfeld, Yaron Berkovich

**Affiliations:** 1 Ruth and Bruce Rappaport Faculty of Medicine, Technion – Israel Institute of Technology, Haifa, Israel; 2 Department of Orthopedic Surgery, Carmel Medical Center, Haifa, Israel

**Keywords:** Nationwide Readmissions Database, navigation-guided surgery, Nationwide Readmission Database, readmission (NRD), total hip arthroplasty

## Abstract

**Background:**

Navigation-guided total hip arthroplasty (THA) has been increasingly adopted to improve implant positioning and early outcomes; however, its population-level clinical and economic impact remains uncertain.

**Methods:**

Using the Nationwide Readmissions Database (NRD) (2020–2022), elective primary THA performed on hospital day 0 was identified in this study. Navigation-guided cases were compared with conventional THA. A 1:5 propensity score–matched cohort was constructed adjusting for demographics, payer, comorbidities, calendar year, and hospital characteristics. Primary outcomes included index hospitalization complications and 90-day readmission with readmission-associated procedural escalation. Readmission resource utilization and modeled 100-case episode-of-care costs were assessed.

**Results:**

Among 366,375 elective THA procedures, navigation use increased from 2.9% (2020) to 4.3% (2022). In the matched cohort (*n* = 77,214), navigation was associated with shorter length of stay and lower rates of intraoperative fracture, blood loss anemia, acute kidney injury, and hip dislocation (all *p* < 0.01). Ninety-day readmission was lower with navigation (3.6% vs. 4.9%; odds ratio 0.72, 95% confidence interval 0.65–0.79), as were early revision and reoperation events. However, index hospitalization charges were higher (+$15,384 per case), and a modeled episode-of-care analysis did not demonstrate cost offset within 90 days.

**Conclusions:**

Navigation-guided elective THA was associated with improved short-term outcomes but higher upfront charges. Because the NRD does not capture surgical approach, implant positioning, implant-specific details, surgeon volume, or longer-term outcomes, these findings should be interpreted as hypothesis-generating associations rather than definitive evidence of a causal technology effect.

## Introduction

Total hip arthroplasty (THA) is widely regarded as one of the most successful and cost-effective procedures in orthopedic surgery (1). Ongoing technical innovation focuses on optimizing implant positioning, restoring biomechanics, and reducing complications to improve early recovery and long-term durability (1). Navigation-guided technology has been increasingly adopted to enhance intraoperative accuracy in component placement and leg-length restoration (2–4). Because even modest deviations in cup orientation, offset, or leg length can contribute to impingement and instability, and because most dislocations occur early, technologies that improve reproducibility have strong theoretical appeal (5).

Despite these proposed advantages, the population-level value of navigation-guided THA remains uncertain. Meta-analyses of imageless and CT-based navigation have reported improvements in radiographic accuracy and/or leg-length discrepancy but have shown mixed findings for dislocation and other clinically meaningful endpoints, highlighting the challenge of demonstrating incremental benefit in an already high-performing elective procedure (2, 3). Registry-scale evaluations similarly report variable associations between computer guidance and revision risk or patient-reported outcomes, emphasizing the need for careful design and risk adjustment (6).

The present study was designed to evaluate whether navigation-guided THA is associated with improved short-term clinical outcomes and to contextualize these outcomes against index hospitalization charges in a national readmissions framework.

Accordingly, we used the Nationwide Readmissions Database (NRD) (2020–2022) to evaluate national adoption trends of navigation-guided elective primary THA and to examine its association with (1) index hospitalization outcomes, (2) 90-day readmission and readmission-associated procedural escalation, and (3) readmission resource utilization. We further performed a modeled 100-case episode-of-care analysis to contextualize short-term economic implications. We hypothesized that navigation-guided THA would be associated with lower 90-day readmission and early escalation events but higher index hospitalization cost (2–4).

## Materials and methods

### Data source

We conducted a retrospective cohort study using the NRD for years 2020–2022. The NRD is an all-payer administrative database developed by the Healthcare Cost and Utilization Project that enables tracking of patient readmissions within a calendar year using unique linkage identifiers. Sampling weights were applied to generate nationally representative estimates. Because the NRD contains deidentified data, this study was considered exempt from institutional review board oversight.

### Cohort identification

Elective primary THA admissions were identified using ICD-10-PCS procedure codes for open replacement of the right or left hip joint. The cohort was restricted to procedures consistent with elective primary THA using synthetic substitute components. A complete list of included ICD-10-PCS codes is provided in Supplementary Appendix Table S1.

To minimize misclassification of non-primary or non-elective procedures, admissions were excluded if they contained diagnosis or procedure codes indicating revision arthroplasty, prosthetic joint infection, osteomyelitis, septic arthritis, fracture-related arthroplasty, periprosthetic fracture, malignancy-related reconstruction, articulating spacer placement, component removal or exchange, or other salvage/staged arthroplasty procedures. Diagnosis- and procedure-based exclusion categories are summarized in Supplementary Appendix Table S2.

To ensure complete 90-day follow-up, index procedures were limited to discharges occurring between January and September of each calendar year, allowing full observation of readmissions within the same year. Discharges without complete 90-day follow-up were excluded from 90-day analyses to prevent incomplete ascertainment of readmission events. In addition, planned admissions for contralateral primary THA were excluded from readmission analyses, as these represented staged elective procedures rather than unplanned postoperative readmissions.

Administrative datasets enable national-scale evaluation of short-term outcomes but require strategies to reduce bias and measurement artifact. Therefore, we restricted the cohort to elective primary THA performed on hospital day 0 to reduce heterogeneity related to delayed operative care and evolving inpatient trajectories. Propensity score methods were used to improve comparability between navigation-guided and conventional THA groups, and balance diagnostics were assessed using standardized mean differences.

### Exposure definition

The primary exposure was navigation-guided THA. Navigation-guided procedures were identified by ICD-10-PCS computer-assisted lower-extremity procedure codes recorded during the index hospitalization, including 8E0YXBZ (a computer-assisted procedure of the lower extremity) and, when separately coded in the NRD code-year files, imaging-specified computer-assisted lower-extremity codes 8E0YXBF, 8E0YXBG, and 8E0YXBH. Eligible THA admissions without these navigation-related codes were classified as conventional THA. Robotic-assisted procedure codes were not included in the navigation exposure definition, unless they also carried a computer-assisted navigation code.

### Outcomes

Outcomes were evaluated at two levels.

*Index hospitalization outcomes* included in-hospital perioperative complications and resource utilization measures, specifically length of stay, total hospitalization charges, intraoperative fracture, blood loss anemia, acute kidney injury, hip dislocation, and other medical complications as defined by standardized ICD-10 coding algorithms.

*Ninety-day outcomes* included the following:

1. All-cause 90-day readmission
2. True component-level hip revision during readmission
3. Hip-related reoperation during readmission
4. Any inpatient procedure recorded during readmission

Among readmitted patients, secondary resource utilization outcomes included readmission length of stay, total readmission charges, and time to readmission.

### Covariates

Baseline covariates included demographic variables (age and sex), primary payer (Medicare, Medicaid, private insurance, other), calendar year, and hospital-level characteristics (bed size, teaching status, and geographic region).

Comorbidity adjustment incorporated conditions relevant to elective arthroplasty risk modeling, including hypertension, dyslipidemia, obstructive sleep apnea, chronic anemia, osteoporosis, type 2 diabetes mellitus, chronic kidney disease, congestive heart failure, chronic lung disease, obesity, peripheral vascular disease, prior cerebrovascular accident, prior myocardial infarction, thyroid disorders, Parkinson disease, and Alzheimer disease.

### Statistical analysis

Baseline characteristics were compared between navigation-guided and conventional THA in the unmatched cohort using appropriate univariate tests.

Because baseline differences were present, propensity score matching was performed to improve comparability between the groups. A 1:5 nearest-neighbor matching strategy without replacement was used. The propensity model incorporated demographic characteristics, payer status, hospital characteristics, calendar year, and comorbidities to achieve comprehensive risk adjustment.

Postmatching balance was assessed using standardized mean differences, with a threshold of <0.1 indicating acceptable covariate balance. All matched variables met this criterion.

Following matching, discharge-level sampling weights were reapplied to generate nationally representative estimates for outcome reporting.

Postmatching outcome analyses were performed within the propensity score–matched cohort after confirming adequate covariate balance. Binary outcomes were analyzed using logistic regression models, and continuous outcomes were analyzed using linear regression models, with model specification selected according to outcome type and distribution. Effect estimates are reported with 95% confidence intervals, including odds ratios for binary outcomes, mean differences for continuous outcomes, and absolute risk differences where clinically informative. A two-sided *p* value <0.05 was considered statistically significant. Statistical analyses were performed using SPSS (IBM Corp., Armonk, NY, USA).

## Results

### Trends in navigation-guided total hip arthroplasty

**Figure 1:**
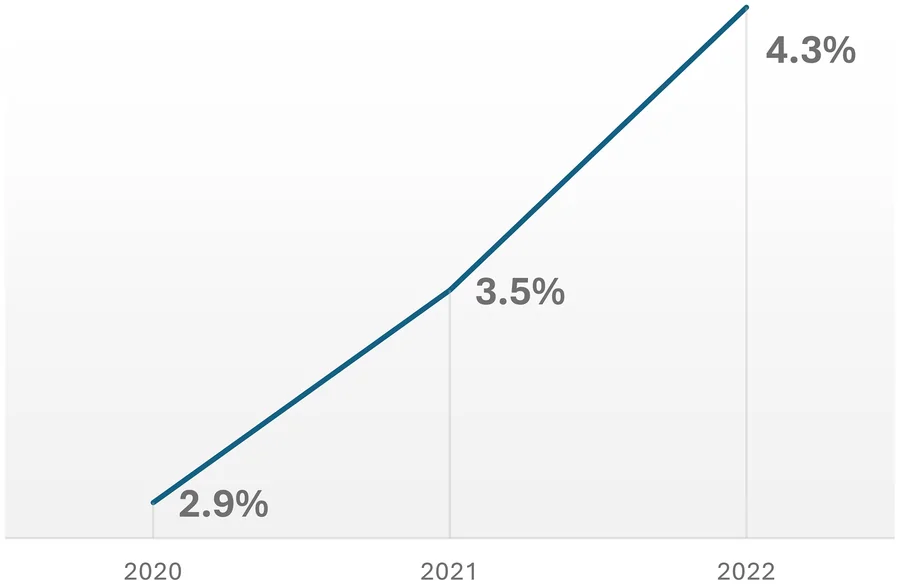
Temporal trends in the utilization of navigation-guided primary total hip arthroplasty, 2020–2022.

Between 2020 and 2022, a total of 366,375 elective primary total hip arthroplasties performed on hospital day 0 were identified. Navigation-guided THA accounted for 3.4% of cases overall. Utilization increased progressively over the study period, rising from 2.9% in 2020 to 3.5% in 2021 and 4.3% in 2022 (*p* < 0.001 for trend) (Figure 1).

### Matched cohort

After 1:5 propensity score matching and reapplication of discharge weights, the final analytic cohort comprised 77,214 admissions, including 12,322 (16.0%) navigation-guided THA and 64,892 (84.0%) conventional THA. Covariate balance was excellent, with standardized mean differences below 0.1 for all matched variables, including patient demographics, payer status, calendar year, hospital characteristics, and comorbidities.

### Index hospitalization outcomes

Index hospitalization outcomes are summarized in Table 1. Mean age was comparable between the groups (67.31 ± 10.70 vs. 67.22 ± 10.60 years, *p* = 0.38). Navigation-guided THA was associated with a shorter length of stay [1.60 ± 1.59 vs. 1.91 ± 2.40 days; mean difference, −0.31 days; 95% confidence interval (CI), −0.34 to −0.28; *p* < 0.001]. Total index hospitalization charges were significantly higher in the navigation cohort than in the other cohort ($87,138 ± 58,130 vs. $71,754 ± 47,614; mean difference, +$15,384; 95% CI, +$14,294 to +$16,474; *p* < 0.001).

**Table 1:** Index hospitalization outcomes following navigation-guided versus conventional elective primary total hip arthroplasty in the propensity-matched cohort.

Outcome	Conventional THA (*n* = 64,892)	Navigation THA (*n* = 12,322)	Effect estimate (95% CI)	*p*-Value
Age (years) mean (SD)	67.22 (10.60)	67.31 (10.70)	MD +0.09 (−0.12 to +0.30)	0.38
Length of stay (days), mean (SD)	1.91 (2.40)	1.60 (1.59)	MD −0.31 (−0.34 to −0.28)	<0.001
Total index charges (USD), mean (SD)	71,754 (47,614)	87,138 (58,130)	MD +15,384 USD (+14,294 to +16,474)	<0.001
Intraoperative fracture (%)	0.9	0.5	OR 0.56 (0.43–0.72); ARD −0.40%	<0.001
Blood loss anemia (%)	15.0	9.7	OR 0.61 (0.57–0.65); ARD −5.30%	<0.001
Acute kidney injury (%)	1.8	1.4	OR 0.78 (0.66–0.91); ARD −0.40%	<0.001
Hip dislocation (%)	0.2	0.1	OR 0.49 (0.27–0.88); ARD −0.10%	0.002

All analyses were performed in the 1:5 propensity-matched cohort with standardized mean differences <0.1 for all matched variables. OR, odds ratio; CI, confidence interval; ARD, absolute risk difference.

Overall in-hospital complication rates were low in both groups. However, navigation-guided THA demonstrated lower rates of intraoperative fracture [0.5% vs. 0.9%; odds ratio (OR) 0.56, 95% CI 0.43–0.72], blood loss anemia (9.7% vs. 15.0%; OR 0.61, 95% CI 0.57–0.65), acute kidney injury (1.4% vs. 1.8%; OR 0.78, 95% CI 0.66–0.91), and hip dislocation (0.1% vs. 0.2%; OR 0.49, 95% CI 0.27–0.88).

### Ninety-day outcomes

Ninety-day outcomes are presented in Table 2. Navigation-guided THA was associated with a significantly lower rate of any 90-day readmission (3.6% vs. 4.9%, *p* < 0.001), corresponding to an odds ratio of 0.72 (95% CI 0.65–0.79).

**Table 2:** Ninety-day readmission and procedural escalation outcomes following navigation-guided versus conventional elective primary total hip arthroplasty in the propensity-matched cohort.

Outcome	Conventional THA (%)	Navigation THA (%)	Odds ratio (95% CI)	*p*-Value	Absolute risk difference (%)
Any 90-day readmission	4.9	3.6	0.72 (0.65–0.79)	<0.001	−1.3
True component-level hip revision	1.0	0.7	0.73 (0.59–0.93)	0.007	−0.3
Hip-related reoperation	1.2	0.8	0.69 (0.56–0.84)	<0.001	−0.4
Any procedure during readmission	3.4	2.3	0.68 (0.60–0.77)	<0.001	−1.1

All analyses were performed in the 1:5 propensity-matched cohort with standardized mean differences <0.1 for all matched variables. CI, confidence interval.

Similarly, navigation-guided THA demonstrated reduced rates of escalation procedures during readmission. True component-level hip revision occurred in 0.7% of navigation cases compared with 1.0% of conventional cases (OR 0.73, 95% CI 0.59–0.93; *p* = 0.007). Hip-related reoperation occurred in 0.8% vs. 1.2% of cases, respectively (OR 0.69, 95% CI 0.56–0.84; *p* < 0.001). Any procedure recorded during readmission occurred in 2.3% of navigation cases compared with 3.4% of conventional cases (OR 0.68, 95% CI 0.60–0.77; *p* < 0.001).

### Readmission resource utilization

Readmission resource utilization is summarized in Table 3. Among patients who were readmitted within 90 days, navigation-guided THA was associated with a shorter readmission length of stay (4.45 ± 4.83 vs. 5.17 ± 5.64 days; mean difference, −0.72 days; 95% CI, −1.21 to −0.23; *p* = 0.01). There were no significant differences in readmission total charges (mean difference, +$7,432; 95% CI, −$2,342 to +$17,206; *p* = 0.13) or time to readmission (mean difference, +1.80 days; 95% CI, −0.71 to +4.31; *p* = 0.16).

**Table 3:** Readmission resource utilization among patients readmitted within 90 days.

Outcome	Conventional THA	Navigation THA	Mean difference (95% CI)	*p*-Value
Readmission total charges, USD, mean (SD)	78,408 (97,191)	85,840 (98,554)	+7,432 USD (−2,342 to +17,206)	0.13
Readmission LOS, days, mean (SD)	5.17 (5.64)	4.45 (4.83)	−0.72 (−1.21 to −0.23)	0.01
Days to readmission, mean (SD)	29.88 (25.42)	31.68 (25.29)	+1.80 (−0.71 to +4.31)	0.16

### Episode-of-care cost modeling (100-case analysis)

To contextualize the clinical and economic implications of reduced readmission rates, a modeled 100-case episode-of-care analysis was performed using observed mean index hospitalization and readmission costs. Although navigation-guided THA was associated with a 1.3% absolute reduction in 90-day readmission (3.6% vs. 4.9%), this corresponded to an estimated reduction of approximately $75,000 in downstream readmission-related expenditures per 100 cases.

However, the higher index hospitalization cost observed in the navigation cohort (+$15,384 per case) translated into an incremental $1.46 million increase per 100 procedures within a 90-day episode-of-care framework. Thus, while navigation was associated with lower readmission incidence and reduced early procedural escalation, these downstream savings did not offset the increased upfront hospitalization charges over the short-term follow-up period.

## Discussion

In this contemporary national analysis of elective primary THA performed on hospital day 0, navigation-guided surgery was associated with lower 90-day readmission and fewer readmission-associated escalation procedures, including true component-level revision and hip-related reoperation (7). These findings were observed in a rigorously propensity-matched cohort with excellent balance across demographic, comorbidity, payer, and hospital-level variables (8). However, navigation was also associated with substantially higher index hospitalization charges, and a modeled episode-of-care analysis did not demonstrate short-term cost offset within 90 days (9).

The reduction in 90-day readmission (3.6% vs. 4.9%) and early escalation events is clinically relevant, as these outcomes represent complications significant enough to require rehospitalization or operative intervention (10). Although absolute differences were modest, the consistency across multiple endpoints strengthens the inference that navigation may contribute to improved early postoperative stability. The associated reductions in intraoperative fracture and early dislocation provide biologically plausible mechanistic support, as improved component positioning and restoration of biomechanics may reduce early instability and technical complications (11, 12).

Prior studies of navigation-guided and computer-assisted THA have most consistently demonstrated improvements in radiographic accuracy, acetabular component positioning, and leg-length restoration, whereas evidence for improvements in patient-reported outcomes, dislocation, revision, or other clinical endpoints remains less definitive (2, 3, 6, 12, 13). The present findings extend this literature by evaluating national 90-day readmission and readmission-associated procedural escalation; however, the observational nature of the NRD precludes conclusions regarding causality or patient-reported benefit.

Nevertheless, interpretation must be cautious. The NRD does not capture surgical approach, implant manufacturer or design, component orientation, leg-length restoration, surgeon experience, annual surgeon THA volume, hospital arthroplasty volume, operative complexity, dysplasia, deformity severity, or enhanced recovery pathway participation. Navigation-guided THA may be preferentially performed at high-volume arthroplasty centers or technologically advanced institutions that inherently achieve better outcomes regardless of navigation use. Therefore, the observed associations may partially reflect center-level, surgeon-level, or pathway-level effects rather than the navigation technology itself. Because approach- and implant-specific variables are unavailable in the NRD, no approach-stratified, implant-specific, or hospital-volume interaction analyses could be performed; related statements have been framed as limitations and hypotheses for future research rather than conclusions supported by the present data.

From an economic perspective, navigation-guided THA was associated with approximately $15,000 higher index hospitalization charges per case (9). Although readmission-related expenditures were modestly lower in aggregate, the 100-case modeled analysis demonstrated that downstream savings did not offset the increased upfront charges within a 90-day framework (14). Although charges are imperfect surrogates for true costs, these findings suggest that short-term economic value remains uncertain and may depend on institutional reimbursement structures and longer-term outcomes not captured in this analysis (14).

Navigation utilization increased steadily between 2020 and 2022, indicating ongoing diffusion into routine practice (8). As adoption expands, identifying patient populations or clinical contexts in which navigation provides the greatest marginal benefit will be essential (13). Future studies incorporating surgical approach, implant-specific variables, and longer-term revision outcomes are needed to better define where navigation-guided THA offers true value in contemporary arthroplasty care (10, 13).

## Conclusions

Navigation-guided elective primary THA was associated with lower 90-day readmission and fewer early revision-level and reoperation events, as well as shorter length of stay, compared with conventional THA. These short-term clinical advantages occurred alongside substantially higher index hospitalization charges, and downstream savings did not offset increased upfront cost within a 90-day framework. Because key operative variables, including surgical approach, implant positioning, implant-specific details, surgeon experience, and institutional arthroplasty volume, are not available in the NRD, these findings should be interpreted as national-level associations requiring confirmation in registry or prospective datasets with longer follow-up.

## Data Availability

Publicly available datasets were analyzed in this study. These data can be found here: https://hcup-us.ahrq.gov/db/nation/nis/nisdde.jsp.
