# Supplementary material for: Navigation-guided elective primary total hip arthroplasty is associated with reduced 90-day readmission and early revision at increased index cost: a national analysis
Source: Front Surg. 2026 Aug 17;13:1818308. doi: 10.3389/fsurg.2026.1818308 (PMC13527035; doi:10.3389/fsurg.2026.1818308)
Supplement: Supplementary file 1 [file Table1.docx]

**Supplementary Appendix Table S1. ICD-10-PCS procedure codes used to identify elective primary total hip arthroplasty**

| **Category** | **ICD-10-PCS codes included** | **Description** |
| --- | --- | --- |
| Right primary THA | 0SR901Z, 0SR902Z, 0SR903Z, 0SR904Z, 0SR906Z, 0SR907Z, 0SR908Z, 0SR909Z, 0SR90AZ, 0SR90BZ, 0SR90JZ, 0SR90KZ | Open replacement of the right hip joint with synthetic substitute components |
| Left primary THA | 0SRB01Z, 0SRB02Z, 0SRB03Z, 0SRB04Z, 0SRB06Z, 0SRB07Z, 0SRB08Z, 0SRB09Z, 0SRB0AZ, 0SRB0BZ, 0SRB0JZ, 0SRB0KZ | Open replacement of the left hip joint with synthetic substitute components |
| Ambiguous codes excluded from the primary THA definition | 0SR90EZ, 0SRB0EZ | These codes were excluded from the final cohort definition to reduce the risk of misclassifying articulating spacer placement, infection-related staged arthroplasty, or salvage procedures as elective primary THA |

**Abbreviations:** THA, total hip arthroplasty; ICD-10-PCS, International Classification of Diseases, Tenth Revision, Procedure Coding System.

Admissions were included only if the index hospitalization was elective and the THA procedure was performed on hospital day 0. Admissions were excluded if diagnosis or procedure codes indicated revision arthroplasty, prosthetic joint infection, osteomyelitis, septic arthritis, fracture-related arthroplasty, periprosthetic fracture, malignancy-related reconstruction, component removal or exchange, articulating spacer placement, or other salvage/staged arthroplasty procedures.

**Supplementary Appendix Table S2. Diagnosis and procedure-based exclusions applied to define elective primary THA**

| **Exclusion category** | **Rationale** |
| --- | --- |
| Revision arthroplasty or component exchange | Excluded to ensure that the cohort represented primary THA rather than revision procedures |
| Prosthetic joint infection, osteomyelitis, or septic arthritis | Excluded to avoid inclusion of infection-related staged arthroplasty, spacer placement, or salvage procedures |
| Fracture-related arthroplasty | Excluded to remove non-elective THA performed for acute femoral neck fracture, acetabular fracture, or other trauma-related indications |
| Periprosthetic fracture | Excluded because these admissions may represent revision, trauma, or complication-related arthroplasty rather than elective primary THA |
| Malignancy-related reconstruction | Excluded to avoid inclusion of oncologic reconstruction or tumor-related arthroplasty |
| Removal, replacement, or revision of hip prosthetic components | Excluded to avoid misclassification of revision or staged procedures as primary THA |
| Articulating spacer placement or staged arthroplasty procedures | Excluded to prevent infection-related staged operations from being classified as elective primary THA |
| Non-elective admission status | Excluded to restrict the cohort to elective primary THA admissions |
| THA performed after hospital day 0 | Excluded to reduce heterogeneity related to delayed operative care, inpatient fracture treatment, evolving complications, or non-elective inpatient indications |
